# Digitalization in Dentistry: Dentists’ Perceptions of Digital Stressors and Resources and Their Association with Digital Stress in Germany—A Qualitative Study

**DOI:** 10.3390/healthcare13121453

**Published:** 2025-06-17

**Authors:** Julia Sofie Gebhardt, Volker Harth, David A. Groneberg, Stefanie Mache

**Affiliations:** 1 Institute for Occupational and Maritime Medicine (ZfAM), University Medical Center Hamburg-Eppendorf (UKE), Seewartenstraße 10, 20459 Hamburg, Germany; juliasgebhardt@gmail.com (J.S.G.); harth@uke.de (V.H.); 2 Institute of Occupational, Social and Environmental Medicine, Goethe University, 60590 Frankfurt, Germany; groneberg@med.uni-frankfurt.de

**Keywords:** digitization, dentistry medicine, dentists, digital stress, medical staff, practices, preventive measures, technostress

## Abstract

**Background**: The digital transformation in dentistry is increasingly reshaping treatment procedures, offering new opportunities and advancements. While digitalization promises enhanced efficiency and quality of care through the standardization, acceleration, and simplification of workflows, it also introduces challenges related to mental health. Studies investigating digitization-associated stressors and resources, as well as health- and work-related outcomes, in the dental sector are still rare. In the context of ongoing digitalization, further studies are needed to examine the need for and the current status of the implementation of measures preventing techno-stress and stress-related outcomes. This study explores the use of digital tools in dental practices and their relationship to the techno-stress among German dentists. It identifies key stressors and resources associated with digital technologies, aiming to inform preventive measures, as well as training and support strategies to mitigate digital stress. **Methods**: A qualitative study was employed, involving ten problem-centered, guideline-based expert interviews with German dentists. The interviews were analyzed using MAXQDA software, following the focused interview analysis framework by Kuckartz and Rädiker. Coding and thematic analysis adhered to the Consolidated Criteria for Reporting Qualitative Research (COREQ) checklist and qualitative research quality criteria by Mayring. **Results**: This study identified a dual impact of digitalization in dentistry. On the one hand, digital tools significantly enhance workflow efficiency, diagnostic accuracy, and patient outcomes. On the other hand, they pose challenges like technostress, high financial costs, and the need for continuous learning. Findings reveal that the perceived usefulness of digital technologies is closely linked to the level of techno-stress experienced, while the amount, intuitiveness, and ease of use significantly influence stress levels. **Conclusions**: Digital transformation offers substantial benefits for dental practices but requires a balanced approach to implementation. Participants highlighted the need for proactive measures, such as targeted training, technical support, and stress-reducing interventions to reduce techno-stress levels. The digital transformation must be supported by coordinated efforts across academia, industry, and policy to strengthen digital competencies—creating a healthier, more resilient digital work environment. Future research should focus on the causal relationship between techno-stress and adverse long-term consequences, such as burnout or mental disorders, among dentists.

## 1. Introduction

Recent years have seen significant advancements in dentistry driven by new digital technologies that promise improved treatment methods and increased efficiency. However, the transition to a digital working environment also brings challenges, particularly regarding the well-being of dental professionals. While digital tools can enhance precision and workflow, they may also increase stress due to continuous learning demands, system malfunctions, and heightened performance expectations. Consequently, the impact of digitalization on mental health—especially stress, strain, and job satisfaction—is receiving growing attention [1].

Digital assistance systems such as intraoral scanners, 3D X-ray devices, telemedicine, and CAD/CAM systems enable comprehensive diagnoses, coordinated treatments, and efficient monitoring. Digital workflows standardize processes, potentially reducing errors and improving outcomes [2]. A survey of German dentists found that 92% expect increased digitalization, with 68% willing to digitalize analogue processes; however, time, costs, data security, and technical knowledge remain barriers [3].

The “digital revolution” is described as simplifying and accelerating dental workflows [4]. Alongside these benefits, the concept of “techno-stress”—first introduced by Craig Brod in the 1980s—has gained attention. Techno-stress refers to difficulties coping with new technologies, leading to symptoms such as fatigue, frustration, and reduced job satisfaction [5,6,7,8]. Five main techno-stress creators have been identified [9]:

- Techno-overload: Pressure to work faster and handle more tasks [9,10,11];
- Techno-complexity: Difficulty mastering complex tools requiring continuous learning [12];
- Techno-uncertainty: Stress from rapid technological changes undermining confidence [13];
- Techno-insecurity: Fear of job loss due to automation [13];
- Techno-invasion: Blurred boundaries between work and personal life due to constant connectivity [13].

If these stressors dominate, they can reduce job satisfaction and well-being. Conversely, techno-stress inhibitors—such as training, technical support, employee involvement, and organizational commitment—can mitigate these effects and improve workplace well-being [8,9].

Recent studies highlight moderate-to-high levels of techno-stress among healthcare professionals, linking stressors to psychosocial demands and burnout risk [14,15,16,17,18,19]. In dentistry, research remains limited but suggests similar patterns, with techno-overload, complexity, and uncertainty as key challenges [20].

The objective of this study is twofold: firstly, to conduct a qualitative investigation assessing the use of digital treatment methods in daily dental practice; and, secondly, to examine their relationship to the experience of techno-stress. In this context, this study aims to identify specific stressors and resources associated with the integration of digital technologies in dentistry. Furthermore, the importance of preventive measures, training, and support options will be examined, with the aim of addressing the current research gap.

During the initial analysis, several key challenges became apparent: a lack of user confidence, insufficient integration of digital tools into existing workflows, and concerns regarding the long-term sustainability of digital transformation. Based on these identified challenges, the following key research questions have been shaped:

1. What are the positive and negative experiences of dentists in using digital assistance systems in clinical practice?
  This question focuses on the individual experiences of dentists, highlighting both the advantages and difficulties they have encountered through digital transformation in their daily work.
2. How does the perceived usefulness, intuitiveness, and ease of use of digital technologies influence the level of techno-stress experienced by dentists?
  This question examines how usability factors affect stress levels and overall acceptance of digital tools.
3. What specific techno-stressors and techno-stress inhibitors are associated with the digitalization of dental practice?
  This question identifies key stress-inducing and stress-reducing factors within digital dental environments.
4. What training formats and support measures do dental professionals consider necessary for effectively integrating digital technologies into practice?
  This question investigates preferred learning methods and support needs for successful and sustainable digital adoption.
5. What strategies are required to ensure a sustainable, ethically sound, and health-conscious implementation of digital technologies in dentistry?
  This question focuses on organizational, policy-level, and ethical aspects that support long-term, responsible digital transformation.

## 2. Materials and Methods

### 2.1. Study Design

A qualitative research approach was adopted for this study. The objective of this approach is to examine and describe complex environments and issues of interaction. In contradistinction to the quantitative approach, it does not aspire to formulate generalizable statements about the subjects under investigation [19]. Instead, the inclusion of the perspective of dentists with their respective experiences from the working environment creates a realistic insight and a detailed understanding [20]. In order to achieve and assess an adequate reporting quality, the COREQ-checklist was used as the basis for the evaluation of the expert interviews [21].

### 2.2. Participant Selection, Inclusion Criteria, and Setting

In order to obtain a representative and relevant sample for this study, a set of inclusion criteria was established to guide participant selection. The inclusion criteria were (1) employment in a dental individual or group practice, clinic or medical care center in Germany (2) for at least 6 months (3) in an employment relationship of at least part-time (≥20 h/week), (4) using digital assistance systems. These inclusion criteria were designed to ensure that the participating dentists possessed substantial long-term experience with digital treatment methods in their respective specialties. Moreover, they contributed to establishing a consistent and knowledgeable participant pool, which was essential for obtaining valid insights into the use and perception of digital assistance systems in dental care settings. To ensure meaningful and targeted data collection, individuals were excluded if they had less than six months of experience in their current position or worked fewer than 20 h per week, as their experiences do not align with the objectives of this study.

Recruitment in Germany was carried out in two different ways: direct contact with dentists by telephone or email, and publication of an information flyer. Recruitment was based on the snowball principle. The initial interviewees were asked to suggest other potential participants. The principle does not pursue the goal of maximizing variance, but is rather oriented toward the structures of the field under investigation. In this way, exhaustive, multi-perspective information on a subject can be obtained [22]. A total of ten dentists took part in this interview study. The number of interviews conducted was determined according to the principle of theoretical saturation. Following the basics of qualitative research, data collection was concluded once it became evident that no further insights emerged from additional interviews. This indicated that sufficient depth and breadth of information had been achieved to comprehensively address the research objectives [23].

### 2.3. Data Collection

Problem-centered, guideline-based expert interviews were used to collect the data. The “expert interview” method was chosen in order to collect the experience and specialized knowledge of individuals with expertise [24,25,26]. The interviews were conducted by J.G. using a prepared guideline to provide orientation and support during the process, ensuring a structured approach to the topic within the broader empirical framework [25,27]. This helped focus attention on specific areas of interest relevant to the research questions [24]. According to Witzel, the interviews followed a problem-centered approach, featuring predominantly open-ended questions to allow participants to share their experiences as freely and openly as possible [28]. The finished transcripts were not sent back to the interview participants for correction.

Questions were asked based on the categories seen in Table 1.

### 2.4. Data Analysis

The further processing of the recorded interviews was divided into two steps: data preparation by transcribing the interviews, followed by data evaluation using focused interview analysis according to Kuckartz and Rädiker (2020) [29]. The verbatim, slightly smoothed transcription of the interviews is based on the transcription rules of Kukartz and Rädiker (2020) [29]. For the data analysis, assessment criteria based on the quality criteria of qualitative research according to Mayring were used to evaluate the quality of processes and results of this research [30]. The central quality criteria of this analysis include a systematic and rule-based approach and the development of a coding guideline as the basis for the evaluation process [30,31]. The qualitative content analysis was conducted in an iterative process in which categories were initially developed deductively on the basis of the theoretical framework and the research questions. In the course of the analysis, the coding system was inductively expanded (see coding system in Table S3). This approach was selected based on initial insights and the emergence of main and sub-categories during the course of data analysis. Both the transcription and the interview analysis were carried out by J.G. using MAXQDA 24 software (VERBI GmbH, Berlin, Germany). To ensure the reliability of the coding, a subset of transcripts was reviewed and verified by a second researcher. Any discrepancies were discussed and resolved by consensus, thereby establishing intercoder reliability.

**Figure 1:**
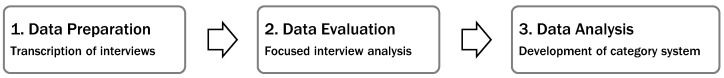
Flowchart of interview processing: from transcription to qualitative analysis.

Figure 1 illustrates the process of interview-data handling, including transcription and qualitative analysis.

### 2.5. Ethics

The confirmation of the ethics application was issued on 5 March 2022 by the Local Psychological Ethics Committee at the Center for Psychosocial Medicine (LPEK) of the University Medical Center Hamburg-Eppendorf (reference number LPEK-0454). The ethical and legal requirements were adhered to throughout the entire course of research. The study participants were informed in a declaration of consent that participation in this study was voluntary and that their personal data would be processed in accordance with data protection guidelines.

## 3. Results

In the following, descriptive results on study participants’ characteristics, as well as main results, are presented. The key findings are outlined based on three main themes: (1) the use and use evaluation of digital assistance systems in dental practices, (2) ethical challenges for dentists using digital assistance systems, and (3) support needs and opportunities in digital dentistry (needs assessment). Table 2 provides an overview of the structure of the Results section, summarizing both the descriptive characteristics of the study participants and the key findings.

Participant quotations are presented to illustrate findings. For readability reasons, a selection of quotations is displayed in the manuscript text; further quotations can be found in Supplementary Materials S1.

### 3.1. Sociodemographic and Occupational Characteristics of the Study Participants

The interview partners fulfilled all the inclusion criteria described above. One interview was conducted in person at the dental clinic, while the others took place via telephone. The interviews lasted an average of 46 min, ranging from 22 and 66 min.

The participant group was diverse in terms of demographic and professional background. The majority were female (60%) practice owners (50%) with a doctoral degree (80%). Around 40% had up to nine years of professional experience working in a general dental practice. The participants ranged in age from 24 to 59 years, with most in their thirties. All participants were of German nationality.

Further details concerning the occupational and sociodemographic characteristics of the participants are provided in Table 3:

### 3.2. Representation of the Use of Digital Assistance Systems

The frequency of digital tools used varies between rarely, irregularly, weekly, and daily. The most frequently used digital technology is practice management software, followed by CAD/CAM, digital X-ray machines, and digital volume technology (DVT). In contrast, intraoral cameras, model scanners, implant planning systems, and various additional management software are used less frequently used in the practices of participants. Table 4 captures the digital tools used in dentistry and their respective fields of application.

### 3.3. Experiences with Digital Assistance Systems in Dentistry: A Practice-Based Evaluation

#### 3.3.1. Negative Experiences in the Use of Digital Assistance Systems

Participants reported various challenges and negative experiences associated with the use of digital assistance systems in dentistry. The statements of the interview participants regarding negative experiences are summarized according to the topic in Table 5.

Thematic analysis of the interview data revealed a series of recurring challenges associated with the use of digital assistance systems in dental practice. The results are structured according to system types, while highlighting overarching themes. Selected quotations are included to illustrate representative experiences rather than isolated perspectives. Patterns were triangulated across participant roles, experience levels, and system types to strengthen interpretive validity.

1. **General Systemic Challenges: Overload, Instability, and Digital Competency**

Participants across all age groups and roles (dentists, dental assistants, and technicians) consistently reported digital overload as a major barrier to effective system integration. This was particularly evident when multiple tools had to be used simultaneously. Time-consuming troubleshooting, unstable software, and non-intuitive interfaces contributed to frustration and inefficiencies. Additionally, many respondents observed that staff often lacked the digital skills necessary for confident system use, impeding the implementation process.

*“There are always things that don’t work, and it can be frustrating when something doesn’t function as it should. It often takes a long time to find the solution.”*

(Participant 7, female, age 30–39)

Such experiences were not isolated; similar sentiments were expressed by participants of varying technical expertise, underscoring that system usability remains a core challenge regardless of digital literacy level. The resulting uncertainty and dependency on external IT support were perceived as disrupting the clinical workflow.

2. **CAD/CAM and Chairside Systems: Efficiency vs. Precision**

Multiple participants, especially clinicians and dental technicians, described CAD/CAM and CEREC systems as resource-intensive rather than time-saving. Both the digital design process and the milling phase were reported to involve frequent errors, repeated recalculations, and machine downtimes. While the potential for in-house production was acknowledged, concerns about restoration fit, aesthetic quality, and material waste were prevalent.

*“It’s not really a time saver. A gold crown still fits best, but that also depends on the material.”*

(Participant 7, female, age 30–39)

*“The computer program was too precise. I had to grind down the teeth quite a lot for the system to recognize the parallelism. A handheld device acknowledges this much sooner and still gets good results.”*

(Participant 7, female, age 30–39)

These views reflect a broader theme identified across the dataset: digital precision, while desirable in theory, may conflict with practical clinical realities—especially in cases where systems require idealized geometries that do not align with natural anatomical variation.

3. **Intraoral Scanners: Limited Applicability and User Dependency**

The efficiency of intraoral scanners was described as highly dependent on the operator’s familiarity with the technology. Several participants indicated that their early scan results were suboptimal until they had adapted their preparation and scanning technique to meet the device’s requirements.

*“My crowns were also, at the beginning, influenced by the preparation, where you first have to get used to the device and figure out what it really needs for the scan and the calculation.”*

(Participant 7, female, age 30–39)

Beyond the learning curve, multiple users highlighted limitations related to clinical conditions. Specifically, the presence of gingival bleeding or subgingival preparations frequently led to inaccurate or incomplete scans.

*“This really only works for patients who do not have gingivitis or where you are not preparing subgingivally. As soon as there is bleeding, it becomes significantly more difficult to scan everything accurately.”*

(Participant 2, female, age 20–29)

This pattern was observed across experience levels and age groups, indicating that it is a device-related constraint rather than a user-specific flaw.

4. **Telematics Infrastructure: High Costs and Low Reliability**

The transition to digital telematics was widely criticized. Participants characterized the systems as immature and the integration process as unnecessarily complex. Frequent malfunctions, high maintenance and acquisition costs, and unclear responsibilities within the system were reported. These problems were particularly concerning to smaller practices with limited IT support.

*“Exactly, it doesn’t work. The technology isn’t fully developed. This router brings constant costs that you don’t want to cover because, honestly, you don’t even want to have it in the first place.”*

(Participant 9, female, age 50–59)

Such views were echoed by both experienced and less experienced users, suggesting that frustration with telematics is widespread and not confined to a specific demographic or professional role.

5. **Practice Management Software: Technical Failures and Data Access**

Numerous participants described recurring problems with practice software, with server instability being the most critical issue. Downtime events led to complete disruptions in daily operations, including the inability to access patient records, schedules, and billing systems.

*“We had disruptions in the server for three days. We didn’t know which patients were coming. We couldn’t input any services. We didn’t know what treatments had taken place before. Nothing was visible in the computer.”*

(Participant 10, female, age 50–59)

Such experiences were reported across both urban and rural practices, with some linking the issue to poor fiber-optic network infrastructure in their region, further highlighting the dependence of digital systems on external connectivity and support.

6. **Implant Planning Software: Visualization and Placement Challenges**

Participants with experience in implantology reported challenges with digital planning systems, especially regarding accurate visualization of the bone structure. Inadequate imaging sometimes leads to suboptimal implant shoulder positioning, affecting the stability of the final placement.

*“In the area of the implant shoulder, there have been cases where it ended up protruding by about a millimeter. Then I couldn’t set it in bone-stable placement. Sometimes, seeing the end of the bone structure can be difficult.”*

(Participant 7, female, age 30–39)

While not mentioned by all participants, this issue was deemed clinically significant by those involved in surgical procedures, indicating a potential risk in implant outcomes when digital planning is based on incomplete anatomical data.

7. **Digital Volume Tomography (DVT): Unequal Access**

While the utility of digital volume tomography was acknowledged, access to the technology was reported as being contingent on patients’ financial means. This was particularly problematic for patients with statutory insurance, where reimbursement was uncertain or unavailable.

*“It’s just another tool that can easily be used with private patients, but with statutory health insurance, there’s always the question of whether patients want to invest in it. It just depends on their income.”*

(Participant 8, female, age 20–29)

This barrier to access illustrates how financial and systemic factors intersect with digital adoption, potentially leading to unequal treatment options for patients based on socioeconomic status.

#### 3.3.2. Positive Experiences in the Use of Digital Assistance Systems

On the other hand, practitioners reported a range of positive experiences regarding the use of digital assistance systems in dentistry. The statements of the interview participants regarding positive experiences are summarized according to the topic in Table 6.

The analysis of positive user experiences revealed several cross-cutting benefits of digital assistance systems in dentistry. These are structured by system type and substantiated with representative quotations. A thematic synthesis was conducted to identify patterns across participants of different age groups, roles (dentists, dental assistants, and technicians), and levels of digital experience. This approach allowed this study to move beyond isolated accounts and highlight shared perceptions of value and functionality.

1. **Overall Perceptions: When Systems Work, They Work Well**

Across all digital system types, a common theme emerged: when technologies function reliably and are properly integrated into the practice environment, they significantly contribute to a smoother, more efficient, and less stressful workflow. Several participants emphasized that successful implementation depends on user acceptance, adequate training, and stable system performance.

*“Fundamentally, this applies to everything: if it works, it’s excellent. If it doesn’t, it leads to frustration.”*

(Participant 5, male, age 30–39)

Participants noted that established systems foster a sense of routine and confidence in daily operations. This positive experience was consistently reported across age groups and roles, suggesting that perceived benefits are not confined to specific user profiles.

2. **CAD/CAM and Chairside Systems: Aesthetic Quality and Efficiency**

The use of CAD/CAM systems—particularly chairside applications such as CEREC—was praised by several dentists and technicians for enabling high-quality, aesthetically pleasing prosthetic restorations. Many participants noted substantial time-savings and increased treatment efficiency, especially when prostheses could be completed in a single session. In addition, the systems were seen as facilitating better communication between dentists and dental laboratories.

*“The good thing is that you can actually complete a complex task within a single session.”*

(Participant 4, female, age 30–39)

Participants from different practice types reported that this technology not only improved clinical workflow but also enhanced patient satisfaction by reducing the number of appointments.

3. **Intraoral Scanners: Versatility and Improved Patient Comfort**

In contrast to some critical views expressed elsewhere in this study, several participants reported consistently positive experiences with intraoral scanners. These users highlighted the reliability of the technology and its wide range of clinical indications, including total prosthetics and implant planning.

Scanners were also praised for improving patient comfort, especially in cases where traditional impressions triggered gag reflexes. Several participants emphasized the potential of digital datasets for simplifying workflows, enabling real-time comparisons, and eliminating the need for physical model storage.

*“Things like that [gag reflex], where patients simply have aversions, especially to an impression. That’s very advantageous.”*

(Participant 3, male, age 50–59)

*“But everything you can do with scanners is huge. Also what the lab can do with the dataset. You no longer have to keep the models; you can use them digitally right away. That’s just enormous.”*

(Participant 9, female, age 50–59)

These positive views were shared across participants with both moderate and extensive digital experience, suggesting growing familiarity and confidence in scanner-based workflows.

4. **Digital Volume Tomography (DVT): Diagnostic Clarity and Surgical Precision**

Participants from various dental specializations identified DVT as a valuable diagnostic and planning tool. The three-dimensional imaging capabilities were seen as providing more accurate anatomical visualization, thereby improving surgical safety—particularly in implant planning, endodontic diagnostics, and sinus or nerve localization.

*“Yes, it is certainly safer. You don’t just have the two-dimensional image to rely on. This means that the maxillary sinus or nerve is surely there where you can see it in the image.”*

(Participant 7, female, age 30–39)

DVT was also noted for its usefulness in identifying apical radiolucencies, lingual concavities in the mandible, retained teeth, and the extent of osteolysis. Its clinical value was consistently acknowledged, despite access and cost constraints discussed elsewhere.

5. **Digital X-Ray Systems: Speed, Communication, and Clinical Integration**

Digital radiography systems were broadly appreciated for their speed, ease of use, and integration into clinical and administrative workflows. Participants reported that instant image access and easy sharing with colleagues or referring dentists improved communication and accelerated treatment decision-making.

*“Digitally, you have it right away. If there’s any doubt, you can quickly call the referring doctor while the patient is still there and say you need the X-ray. So that’s a huge advantage.”*

(Participant 1, male, age 30–39)

*“I think that’s quite good as well, especially when you want to measure the length again during an endo treatment or see what the root looks like.”*

(Participant 2, female, age 20–29)

These insights were consistent across participants from both general practice and specialized disciplines, such as endodontics, suggesting broad clinical relevance.

6. **Practice Management Software: Organization, Documentation, and Transparency**

Despite technical challenges noted elsewhere, participants described several key benefits of practice management software. The systems were credited with streamlining administrative tasks such as time tracking, personnel documentation, and hygiene index monitoring. Several users emphasized the value of digital patient records in simplifying case reviews and ensuring consistent documentation quality.

*“It also creates good documentation and provides security that working hours are recorded clearly and accurately.”*

(Participant 3, male, age 50–59)

*“You can get things done more quickly, and you’re more organized than if you had everything on paper and were carrying around a stack of files. You’re more flexible, which I like.”*

(Participant 2, female, age 20–29)

These advantages were noted across age groups, indicating that administrative digitalization can yield tangible benefits regardless of user-age demographic.

7. **Communication Tools: Flexibility and Real-Time Coordination**

Participants reported that team communication tools, such as Microsoft Teams, improved internal coordination and enabled asynchronous communication—especially in larger practices with multiple treatment rooms or staggered shifts.

*“We are using Microsoft Teams as a team communication tool. This allows you to reach any employee from anywhere in the practice at any time of day.”*

(Participant 5, male, age 30–39)

The use of such systems was perceived as enhancing practice-wide responsiveness and supporting efficient team management.

### 3.4. Stress-Inducing Factors: Competitive Pressures and Strategic Adaptions in the Context of Digital Transformation

Digital transformation in dental practices is shaped not only by technological developments but also by competitive dynamics and strategic positioning. Participants described a wide spectrum of responses to perceived market pressures, ranging from urgency and innovation to detachment and strategic restraint. These varied responses were influenced by factors such as patient expectations, peer benchmarking, geographic location, and existing levels of digital maturity.

**Figure 2:**
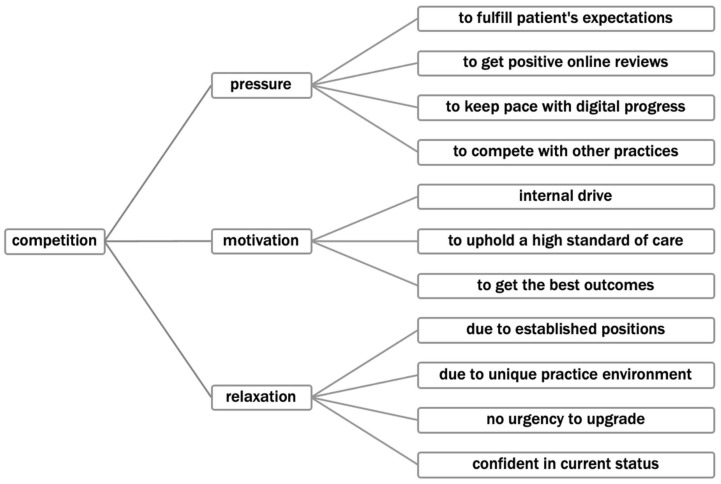
Overview of strategic adaptations in the context of digital transformation.

Figure 2 illustrates the role of competition in shaping various dynamics within dental practices, according to the participant’s answers. It effectively conveys the multifaceted impact of competition on practices, ranging from pressure to motivation and even relaxation, depending on their circumstances and strategic positioning.

Across interviews, a recurring theme was the belief that staying digitally current is essential for long-term viability. Several participants emphasized that falling behind in digital adoption could lead to reputational damage, staff demotivation, and, ultimately, economic decline.

*“Those who do not advance digitalization in their dental practices will face low patient numbers. These practices will not have motivated employees, and, in the long term, they will be at a clear competitive disadvantage. Such practices will ultimately have to close.”*

(Participant 5, male, age 30–39)

Participants working in competitive urban settings, in particular, reported experiencing external pressure to implement the latest digital tools. This pressure was driven by patient demands, online review platforms, and peer comparisons. The presence of digital systems in neighboring practices often served as both a benchmark and motivator, reinforcing the need to modernize in order to remain competitive.

*“People spread the word quickly, saying that only certain doctors had this device and that it worked so much faster. So, external pressure builds up, making you feel like you have to keep up with progress.”*

(Participant 1, male, age 30–39)

While some viewed this pressure as stressful, others perceived it as constructive and motivating—an opportunity to improve care quality and streamline operations. This nuanced interpretation was especially common among younger practitioners, who framed competition as a catalyst for innovation rather than a threat.

*“I wouldn’t describe it as negative pressure. In the end, it makes sense. The things being developed are intended for improvement. Especially in diagnostics and workflow acceleration, it’s a good kind of pressure.”*

(Participant 6, female, age 20–29)

In contrast, other participants—particularly those in rural or less saturated markets—described minimal-to-no competitive pressure. These practices often reported already being well-equipped relative to peers, which fostered a sense of confidence and reduced the urgency to continuously upgrade.

*“I actually don’t know any practice that’s better equipped. And that makes me feel incredibly relaxed about this topic every day.”*

(Participant 5, male, age 30–39)

This divergence highlights how the perceived need for digital transformation is highly context-dependent. Practices in dense, competitive environments tend to frame digital investment as a necessity, while those in more isolated or well-resourced settings may view it as optional or incremental. These findings suggest that strategic adaptation to digitalization is not solely based on technological readiness but is also deeply informed by localized competitive landscapes and individual practice identities.

### 3.5. Ethical Challenges in the Use of Digital Assistance Systems in Dental Practices

The integration of *digital assistance systems* in dentistry has introduced new ethical considerations. These relate primarily to responsibility attribution in the context of errors, data protection, and broader concerns about over-treatment and depersonalization of care.

#### 3.5.1. Responsibility and Error Attribution in Digital Workflows

A central ethical concern involves the extent to which responsibility shifts when digital systems are used. The majority of participants emphasized that responsibility ultimately remains with the dentist, particularly regarding oversight, clinical judgment, and the correct application of digital tools. There was broad agreement that technology should be seen as an aid, not a substitute for professional accountability.

*“No, I know that it ultimately falls on me. I’m the one who was behind the scenes making the preparations. The computer doesn’t make mistakes in that sense; any errors stem from my work.”*

(Participant 7, female, age 30–39)

Participants consistently highlighted the need for active monitoring and professional supervision, especially when delegating digital tasks. While systems may introduce new efficiencies, they do not eliminate the ethical obligation to ensure quality and safety.

Several interviewees noted that digital workflows can facilitate quicker and more targeted error correction. For example, faulty scan sections can be easily re-acquired without compromising the entire dataset. However, others warned that errors may become more difficult to trace within digital workflows, as increased automation and delegation may obscure individual accountability.

*“It’s much easier to correct a mistake. For example, if a tooth isn’t scanned correctly, you can just delete that part and redo it without ruining the entire impression.”*

(Participant 9, female, age 50–59)

*“When more errors creep in, it becomes much more anonymous. It’s harder for the team to identify whether the error occurred at reception, in the treatment room, or with the practitioner.”*

(Participant 8, female, age 20–29)

These observations reflect a broader concern that as digital tools streamline workflows, they may also reduce transparency in where and how errors arise.

#### 3.5.2. Broader Ethical Implications: Data Protection, Over-Treatment, and Depersonalization

Beyond error management, participants raised other ethical concerns stemming from the digitization of dental care. Data protection emerged as a key issue. Multiple respondents noted that safeguarding patient privacy is more complex in digital environments, requiring constant vigilance and additional administrative tasks. Screens visible to patients or visitors were described as potential sources of inadvertent data exposure. One participant recounted a prior cybersecurity incident that prompted costly investments in firewalls and ongoing data protection protocols.

*“We have semi-annual consent forms that patients must sign. Additionally, one must ensure that incoming patients don’t see the X-rays of the previous patient on the screen.”*

(Participant 8, female, age 20–29)

Another ethical dilemma involved the potential for over-treatment. Advanced imaging technologies now enable earlier diagnosis of minor or subclinical findings. While this capability can benefit preventive care, several participants cautioned that it may also lead to unnecessary interventions, especially when clinical thresholds are not clearly defined.

*“There might be a tendency to treat people much sooner because we can identify issues more readily in imaging. I believe we need to find a good balance here.”*

(Participant 1, male, age 30–39)

Finally, participants expressed concerns about the standardization of workflows through digital systems. While this can promote efficiency and consistency, some feared it could also reduce personalization in patient care. The challenge, as described, lies in maintaining the human aspects of dentistry amid increasing automation.

Taken together, these findings highlight how the ethical landscape of dental practice is evolving alongside digital transformation. Dentists are not only adapting technically, but also navigating new responsibilities related to transparency, data protection, patient autonomy, and professional judgment.

### 3.6. Support Needs and Learning Methods for Successful Digital Adoption

The transition to digital assistance systems in dental practices is not only a technical or financial challenge but also an educational and infrastructural one. This section synthesizes interviewee insights regarding prerequisites for successful digital adoption and the types of support necessary to facilitate this transformation.

#### 3.6.1. User-Friendliness and Application Support

A recurring theme across interviews was the need for digital systems to be intuitive and user-friendly. Participants noted that complexity often deters use, whereas streamlined and clearly structured tools encourage frequent application. Particularly in high-stakes and time-sensitive environments such as dental practices, systems must align with clinicians’ workflows rather than requiring time-intensive adaptations.

*“It should not be a technical gimmick. We are not specialists in usability. This is not a pastime in the practice; providing care for patients is our goal.”*

(Participant 10, female, age 50–59)

Beyond usability, participants emphasized that better integration between systems would enhance user experience. The lack of open interfaces was cited as a barrier, leading to fragmented data management and redundant work processes.

*“I would certainly like to see the systems working more integratively with each other. The software interfaces should become more open.”*

(Participant 5, male, age 30–39)

These statements reflect a broader consensus that ease of use and system compatibility are critical enablers of digital adoption.

#### 3.6.2. Willingness to Learn and Age-Related Differences

Willingness to engage with digital tools was strongly associated with both age and previous exposure to technology. Younger practitioners were generally more confident and eager to explore digital options, often citing their university training as a key enabler. In contrast, some more experienced professionals described skepticism toward untested technologies and a preference for approaches already validated in clinical practice.

*“I’m happy to embrace what is proven, scientifically validated. What companies throw on the market that isn’t backed by research, I prefer to stay away from.”*

(Participant 8, female, age 20–29)

However, age alone was not the sole determinant. Several participants—regardless of age—described high levels of motivation, especially when new tools demonstrated tangible clinical benefits or aligned with their personal interest in technology.

*“Digital work motivates me. I’m definitely willing to acquire new skills to make the most of the technical possibilities.”*

(Participant 5, male, age 30–39)

Others acknowledged that learning new systems required patience, persistence, and a readiness to endure initial setbacks—described by one participant as a “valley of tears”—before full utility could be realized.

Importantly, the resistance to digital adoption was often rooted not in age, but in entrenched practice habits and organizational culture. Participants emphasized the importance of moving beyond the “we’ve always done it this way” mentality, which they saw as stifling innovation and progress.

#### 3.6.3. Support Measures for Sustainable Digital Implementation

**Figure 3:**
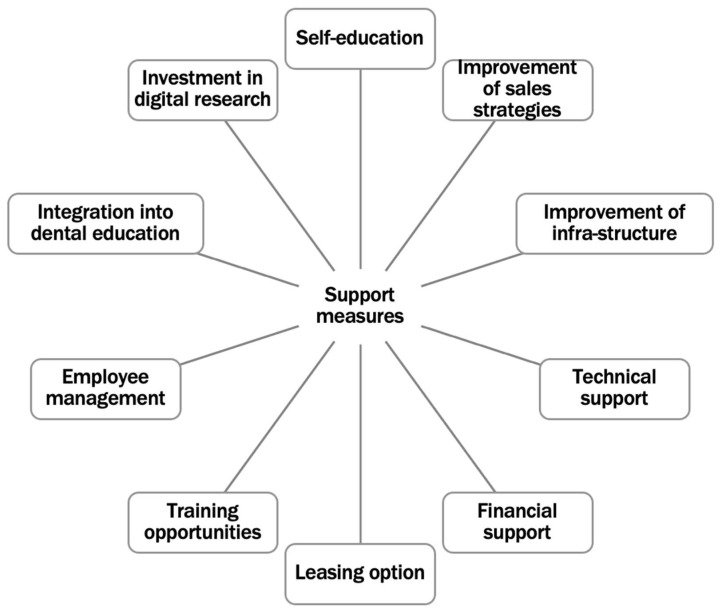
Support measures in the context of digitalization processes.

Participants outlined a range of support mechanisms necessary to facilitate the adoption of digital tools, including infrastructure, education, financial support, and improved communication between manufacturers and end-users. In Figure 3, the potential support measures in the context of digitization processes in dentistry are listed.

At the individual level, self-directed learning was described as essential. Participants consistently reported that they had to independently acquire knowledge about system functions and integration due to the absence of centralized guidance.

*“I have to educate myself. If I’m interested in a topic, I can find information, but I have to work through it all on my own.”*

(Participant 7, female, age 30–39)

Reliable technical support and responsive customer service were highlighted as major gaps. Delays in resolving technical issues and poor support from vendors were seen as major hindrances to daily operations. Additionally, participants expressed a need for better after-sales support and ongoing assistance with maintenance and system updates.

Several practitioners reported that current network infrastructure—especially in rural areas—remains inadequate for modern digital demands. A lack of bandwidth or outdated hardware was seen as a root cause for digital stagnation in some practices.

*“Digitalization hasn’t progressed much because the necessary conditions simply aren’t in place. There are quite a few prerequisites that need to be met.”*

(Participant 2, female, age 20–29)

Financial support also emerged as a core issue. High upfront investment costs and recurring expenses for upgrades, licenses, or training were seen as major barriers—particularly for smaller practices. Participants suggested leasing models, subsidies, and targeted government funding as potential enablers.

*“If the government mandates certain technologies, they also need to support practices financially. Not every practice can afford such upgrades.”*

(Participant 2, female, age 20–29)

*“Leasing is a good solution because technology evolves so quickly. Buying a device often locks you into outdated technology.”*

(Participant 10, female, age 50–59)

From an organizational perspective, internal training and employee management were emphasized as essential. Tailoring training programs to the individual digital literacy levels of staff was seen as a way to increase engagement and reduce resistance.

*“It’s simply a matter of employee management. You need to know which staff members are digitally literate and tailor the training accordingly.”*

(Participant 5, male, age 30–39)

Lastly, there was widespread support for stronger integration of digital competencies into dental education. Universities were encouraged to embed digital technologies into their curricula to ensure that future practitioners enter the field with the necessary skills. At the same time, several interviewees called for more research and academic exploration of digital dentistry to generate robust evidence for its benefits and limitations.

*“I believe dentistry is weak in scientific research. I would appreciate it if universities invested more in digital advancement.”*

(Participant 8, female, age 20–29)

## 4. Discussion

This study aimed to enhance our understanding of how digital treatment methods are used and assessed in the context of everyday dental practice. With regard to the first component of the research’s objective, this study is significant for its thorough investigation of the advantages and potential risks associated with the digitalization of dental practices. While other studies have already elaborated on the effects of digital technologies in various medical fields [8,15,16,32,33,34], this study examined the connections between dentists’ perception of the usefulness of digital technologies and their experience of techno-stress. In relation to the second component of this study’s objective, specific digital stressors and inhibitors that contribute to techno-stress in dentistry were investigated. The needs assessment facilitated the identification of a broad spectrum of support requirements that are essential for the development of a health-promoting digital environment.

### 4.1. Dentists’ Experiences with Digital Assistance Systems: Usage Behavior and Subjective Evaluation in Clinical Practice

The analysis of the interviews displayed several key insights into the utilization of various digital technologies in dentistry and the subjective evaluation thereof by dental practitioners. The dentists interviewed indicated that they utilize digital technologies with considerable frequency. However, a discrepancy in the frequency of utilization among the digital assistance systems was also observed. The dentists reported a range of experiences with digital assistance systems, which were then evaluated.

In comparison to a Dutch study, the frequency of digital technology use among the interviewed dentists in Germany is similar. According to van der Zande et al., Dutch dentists use an average of 6.3 ± 2.3 technologies, with administrative technologies generally being used more frequently than clinical ones [35]. Similarly, our interview study found out that the majority of participants also use administrative technologies, such as practice management software or practice communication software. In contrast, clinical technologies like intraoral scanners and digital three-dimensional X-rays are used less frequently on average. Both studies demonstrated a correlation between usage behavior and the user’s age, as well as the size of the practice.

Digital assistance systems were recognized for their potential to streamline workflows, particularly when systems are well-implemented. Technologies like CAD/CAM and CEREC systems were praised by the participants for time-saving processes, allowing for faster delivery of dental prostheses and enhancing communication with technicians. The study by Jafarpour et al. (2024) similarly concluded that digital fabrication of complete dentures using CAD/CAM leads to cost- and time-savings [36]. However, no advantages were observed in regard to the quality or the number of adjustment visits. This study’s findings showed that intraoral scanners are valued for their reliability, versatility, and the immediate usability of digital data. Pullishery et al. (2023) concluded in their study that the digital impression technique surpasses the conventional impression technique with its advantages [37]. Similar to this study’s findings, they discovered that the use of an intraoral scanner could achieve higher time efficiency and accuracy levels, as well as a more favorable patient perception. Additionally, this study’s interview participants reported that tools like DVT and digital X-rays offer superior imaging capabilities, contributing to better diagnostic outcomes and surgical planning. Another study by Mengel et al. (2005) concluded that periodontal defects could only be detected in two planes with 2D imaging, whereas the 3D images allow for a true-to-scale representation of the bone structures in three planes, without overlap or distortion [38]. Similar to this study’s reports, the DVT scans demonstrated the best imaging quality.

### 4.2. Technology-Associated Stressors and Resources

Based on Ragu-Nathan’s concept of “techno-stress,” the treatment providers’ experiences with digital assistance systems can be categorized into techno-stress creators and techno-stress inhibitors [5]. Techno-stressors in dentistry can include various aspects that increase the stress level of dentists. Similar to other research formats, our study showed that the introduction of new digital systems often leads to a higher stress experience [8]. Table 7 provides an overview of the key stressors mentioned with their respective causes.

In the following, the different stress factors are examined in more detail and classified with the comparative literature.

**Techno-overload** was one of the main stress factors experienced by the surveyed dentists. Due to managing multiple tasks simultaneously, dentists have the general perception of being forced by technology to work faster and more efficiently. Compared to findings of other research formats, this study’s participants similarly reported that this can lead to additional stress when demands are high [5,32]. Consequently, constant techno-overload can be identified as a significant barrier to the implementation of digital assistance systems in dentistry [34,39,40,41]. Another stress factor mentioned by the participants is the pressure to keep up with rapid technological developments. As a comparable study by Bernburg et al. has already shown, competitive pressure can arise among dentists, and it can lead to feelings of inadequacy and stress [18].

Additionally, the interview participants identified **techno-uncertainty** caused by a lack of digital skills as a digital stressor. Specific technologies, including CAD/CAM systems, intraoral scanners, and practice software, have been identified as being dependent on user experience and skills. This study shows that a lower digital competence can be a barrier to the successful implementation of digital technologies. Low digital competence can be rooted in past negative experiences or a lack of knowledge and skills, as the findings of Kuek and Hakkennes (2020) showed [42]. These results support similar findings of other studies in which users who have little contact with digital technologies show higher levels of techno-stress because they lack opportunities to adapt and develop their own skills in using them [43]. In particular, older practitioners who did not grow up with digitalization reported more frequent difficulties in familiarizing themselves with digital processes. Our findings support the study by Gimpel et al. (2018), which found that younger employees, as digital natives, are less prone to techno-stress, while media literacy might be lower among older employees [14].

These issues are often further exacerbated by the complex, sometimes non-intuitive nature of digital work processes; another stress factor which occurs is **techno-complexity**. Tajirian also confirmed this in his study, in which he identified the intuitiveness and usability of the technology, as well as workflow issues, as specific challenges for medical professionals [15]. Additionally, this study’s research findings showed that the challenges experienced by the users include time-consuming troubleshooting and system instability. Especially when technical issues arise, such as network failures or the loss of digital patient data, the workflow can be disrupted, resulting in even more stress. Sommovigo et al. (2023) confirmed in his study that a disruption of the workflow caused by technical problems can cause additional stress for the user [44]. Other findings showed that software errors, equipment failures and technical problems can cause additional stress, especially if there is a lack of technical support [18].

### 4.3. Ethical Challenges for Dentists in the Use of Digital Assistance Systems

The findings on the topic of “ethical challenges” primarily focus on the stressors of **techno-insecurity** and **techno-invasion**, exploring potential fears of loss of control or responsibility by the increasing “robotization” at dental practices. In contrast to a comparable study, none of this study’s interview participants reported a loss of control or responsibility due to digital technologies in the dental context [45]. Instead, the importance of maintaining oversight over digital processes, delegated tasks, and the proper use of digital tools was highlighted by several participants. They emphasized that ultimate accountability remains with the dentist, citing that human intelligence still surpasses technological capabilities. The interview study, however, revealed that protecting patient data can be challenging when working with digital systems. Other studies have also concluded that data protection regulations can create additional pressure [46,47]. Another challenge mentioned by the interviewed dentists was over-treatment and reduced personalization caused by digital transformation. This has not yet been confirmed by any comparable study.

### 4.4. Strengths and Limitations

A notable strength of this study is the meticulous documentation of the participants’ subjective experiences. The qualitative research approach allows for an in-depth exploration of individual perceptions and contextual factors. Unlike quantitative research, which focuses on measuring variables and producing statistically generalizable results, this study’s approach aims to capture the participants’ subjective experiences. The open structure of the interviews engenders a flexible research process. This adaptability facilitates the collection of information that might not have been previously considered.

However, this study is not without its limitations. The qualitative approach precludes generalizations of the results due to the limited number of participants, as it hinders the analysis of potential differences in technostress levels between specific groups. It is therefore recommended that future studies include a greater number of participants to enhance the validity of the findings and allow for more detailed subgroup analyses. Additionally, the analysis was conducted by a single researcher, which may have introduced individual bias in the interpretation of the interview data. This limitation could affect the reliability of the findings, as multiple analysts might have provided broader perspectives and enhanced interpretive validity. In keeping with the open structure, the interview guide did not explicitly name specific techno-stressors. Instead, guiding questions were used to lead the conversation toward potential stressors, as the interviewees were initially expected to identify stressors independently without bias. Another limitation of this study is the lack of longitudinal data to demonstrate a causal relationship between techno-stress and adverse long-term consequences, such as burnout or mental disorders, among dentists. Therefore, research is needed to address these issues in the future.

### 4.5. Implications for Further Research

While digitalization in dentistry has already demonstrated its potential to enhance patient care, its full integration into clinical practice requires ongoing research. By addressing mental health challenges, ethical concerns, and coping mechanisms, future studies can help ensure that digital advancements benefit both clinicians and patients—without compromising well-being. Therefore, future research should focus on several key areas:

As digital tools become increasingly integrated into daily practice, it is crucial to study their impact on the mental and emotional health of practitioners. One important topic for research is digital fatigue, which is becoming more common in an increasingly digitized workplace. Studies should examine how continuous screen time and constant interaction with digital tools affect the cognitive and emotional resilience of dental professionals.

Additionally, it is essential to study how digitalization impacts the patient–dentist relationship. While technology can improve patient engagement and treatment outcomes, it might also create distance by reducing face-to-face interactions. Some patients may even perceive that technology is replacing human care. Furthermore, concerns about data security and patient privacy mentioned by several participants may affect patients’ trust in digital tools, highlighting the need for transparent data governance and ethical implementation. Understanding these effects is crucial to ensuring that digital tools enhance, rather than weaken, the personal connections that make dentistry fulfilling for many practitioners.

Future research should also analyze potential differences between specific groups, for example, gender patterns in digital adoption and techno-stress perception, as initial indications suggest that these experiences may differ across demographic groups. Such an analysis could provide valuable insights into how digitalization affects individuals differently within the dental profession.

### 4.6. Implications for Practice, Policy, and Dental Education

Sustainable digital transformation in dentistry requires not only technological advancements but also strategic organizational approaches to manage techno-stress and foster digital competence. This study underscores that user-friendliness and seamless integration of digital tools into clinical workflows are vital to reduce workload and increase acceptance. Persistent barriers, such as low user confidence and digital uncertainty, highlight the urgent need for intuitive software solutions that minimize training demands and ease onboarding.

To address diverse learning needs and promote effective adoption, dental practices should implement flexible training programs—including individual, group, digital, and hands-on sessions—tailored to different user preferences and skill levels. Embedding digital competencies early in dental education is crucial to cultivating a culture of openness and adaptability, ensuring future professionals are prepared for evolving technologies.

From a policy perspective, coordinated efforts are needed to provide financial and infrastructural support for dental practices, enabling investments in user-centered digital systems and comprehensive training. Moreover, ethical challenges related to data security and patient privacy must be addressed proactively. Clear guidelines, robust cybersecurity measures, and ongoing staff education on data protection are essential to safeguard sensitive information and maintain patient trust in the digital age.

Together, these measures can create a resilient dental workforce equipped to navigate the complexities of digital transformation, improve care quality, and mitigate the negative impacts of techno-stress at organizational levels.

## 5. Conclusions

Digital transformation in dentistry is fundamentally reshaping not only technology and workflows, but also the well-being of dental professionals. The key to successful, stress-reduced implementation lies in targeted training, reliable technical support, and proactive prevention strategies. To fully harness the benefits of digital innovation and safeguard practitioner health, coordinated efforts from academia, industry, and policymakers are essential.

## Figures and Tables

**Table 1:** Thematic categorization of the interview questions.

Topic	Category	Subcategory
1: Sociodemographic and occupational characteristics of the participants	Practice-specific information data	Practice setup
		Dental laboratory
	Person-specific information	Specialization/treatment spectrum
		Work schedule
		Professional career
		Employment contract
		Daily work routine
		Age
		Level of education
2: Use and use evaluation of digital assistance systems in dental practices	Usage behavior	Digital assistance systems that are used
		Usage behavior
		Frequency of use
	Subjective evaluation of use	Positive experience reports
		Negative experience reports
3: Ethical challenges of the use of digital assistance systems in dental practices	Ethical challenges	Responsibility
		Dealing with errors
		Ethical questions
4: Support needs and opportunities in digital dentistry	Needs assessment	Support measures
		Willingness to learn
		Support, maintenance
		User-friendliness of digital tools

**Table 2:** Presentation of themes and subthemes of the key findings.

Theme	Subtheme
Sociodemographic and occupational characteristics of the participants	
Use and use evaluation of digital assistance systems	Representation of the use of digital assistance systems Experiences with digital assistance systems: A practice-based evaluation - Negative experiences - Positive experiences
Stress-inducing factors	Competitive pressures and strategic adaptations
Ethical challenges in the use of digital assistance systems	Responsibility and error attribution in digital workflows Broader ethical implications: data protection, over-treatment, and depersonalization
Support needs and learning methods for successful digital adoption	User-friendliness and application support Willingness to learn and age-related differences Support measures for sustainable digital implementation

**Table 3:** Characteristics of study population.

Category	Characteristic	Frequency (n)	Percentage (%)
Gender	Female	6	60
	Male	4	40
Age	20–29	3	30
	30–39	4	40
	40–49	0	0
	50–59	3	30
Position	Assistant dentist	2	20
	Employed dentist	3	30
	Practice owner	5	50
Profession	Approbation	2	20
	Promotion	8	80
Years of work experience	1–9	4	40
	10–19	3	30
	20–29	2	20
	30–39	1	10
Working pensum (%)	0–19	0	0
	20–39	0	0
	40–59	1	10
	60–79	0	0
	80–100	9	90
(Practice-)Specialization	Implantology/dental surgery	2	20
	Cranio-mandibular dysfunction (CMD)/ functional diagnostics	2	20
	Endodontics/parodontology	2	20
	General	4	40

**Table 4:** Overview of the applied digital tools with their respective fields of application.

Digital Tool	Application
Intraoral scanner	Fabrication of prosthetic restorations, e.g., crowns, interim splints (especially posterior region, all zirconia), and splints Implant work (abutments, crowns, and surgical guiding templates) Documentation for research, e.g., volume change
Digital X-ray	Endodontics, caries diagnostics, and periodontology
Intraoral camera	Visualization of dental plaque, caries, and tooth fractures (diagnosis and patient communication)
Model scanners	Scanning plaster models (prosthetics)
DVT	Pre-surgical baseline assessment (wisdom tooth extraction and cyst exposures) and implant planning
Practice management software	Staff management, communication, quality control (efficient operations)
Online training tools	Continuous professional development (accessible ongoing training)
Patient education software	Treatment plan and consent form signing (enhancing patient interaction)
CAD/CAM technologies	Planning and production of dental prostheses (skill-dependent outcomes)
Digital facebows	Recording and evaluating temporo-mandibular joint (TMJ) trajectories (precise prosthetic design)
3D printers	Producing dental models (streamlined fabrication and improved efficiency)

**Table 5:** Negative experiences reported by the participants in the use of digital assistance systems.

Negative Experiences	Details
Technical problems	Malfunctions, handling difficulties
	Extensive error analysis
	Unstable networks
	Risk of cyber-attacks
	Poorly suited IT infrastructure
Low user friendliness	Reliant on the user’s experience
	Systems’ potential not fully realized
	Non-intuitive workflows
	Lack of interoperability between different digital systems
Clinical documentation	High traceability and controllability
Increased workload	Strict data protection measures
	Need for additional analog safeguards
	System monitoring/controlling
	Staff training
	Transition process
Training	Insufficient training for staff
Digital transformation	Further complications due to the transition process
	Undefined areas of responsibilities
Costs	High substantial expenses for acquisition, maintenance, research and development
	Rapid obsolescence of older devices, necessitating new purchases, increasing financial burdens
Results	Inaccuracy of results in some cases
	Limited indications
	High substance removal

**Table 6:** Positive experiences reported by the participants in the use of digital assistance systems.

Positive Experiences	Details
Availability of information	Enhancement of the availability of information Quick and simultaneous accessibility to patient data across all computers
General reduction of workload and time-savings	Improved communication with dental labs and staff Immediate use of data sets No need for physical storage and archiving Easier delegation of tasks Standardization of procedures Increased efficiency and safety for practitioners Comfortable handling Good integration of the software into existing workflows
Transparency	Better readability and a more comprehensive overview of patient data Ensuring transparent documentation Easy comparability of records Enhancement of the accuracy and clarity of information
Results	High aesthetic outcomes, particularly in prosthetics Excellent visualization of structures, e.g., through DVT images Wide range of indications Minimization of contamination risks by reducing the need for paper records
IT support	Technical support available from software companies
Patients	More comfortable experience during treatments Positive impact on communication between the dentist and the patient Fostering better understanding and trust

**Table 7:** Overview of key stressors with their respective causes.

Techno-Stressors	Causes
Techno-overload	Management of multiple tasks at the same time
	Own claim to work faster and more efficiently
	Competitive pressure
	Rapid technological developments
Techno-uncertainty	Lack of digital skills
	Familiarization with digital work processes
	System instability
Techno-complexity	Complex, non-intuitive digital work processes
	Software errors, equipment failures, technical problems
	Time-consuming troubleshooting
	Lack of technical support
	Dependency on technology
Techno-insecurity	Risk of inadequate data protection/higher data protection regulations
	Risk of over-treatment
Techno-invasion	Loss of control or responsibility
	Reduction of personalization

## Data Availability

The datasets analyzed during the current study are not publicly available due to German national data protection regulations but are available from the corresponding author on reasonable request.

## Supplementary Materials

The following supporting information can be downloaded at https://www.mdpi.com/article/10.3390/healthcare13121453/s1, Table S1: Additional interview quotes; Table S2: COREQ checklist (Consolidated Criteria for Reporting Qualitative studies: 32-item-checklist); Table S3: Coding System Hierarchical Structure.

## Abbreviations

The following abbreviations are used in this manuscript:

MAXQDA	Max Weber Qualitative Data Analysis
COREQ	Consolidated Criteria for Reporting Qualitative Research
CAD/CAM	Computer-aided design/Computer-aided manufacturing
IT	Information Technology
LPEK	Local Psychological Ethics Committee
CMD	Cranio-mandibular dysfunction
DVT	Digital volume technology
TMJ	Temporo-mandibular joint
CT	Computed tomography
AI	Artificial intelligence

## References

[1] Neuhaus A., Lechleiter P., Sonntag K. (2016). Measures and Recommendations for Healthy Work Practices of Tomorrow (MEgA).

[2] Odone A., Buttigieg S., Ricciardi W., Azzopardi-Muscat N., Staines A. (2019). Public Health Digitalization in Europe: EUPHA Vision, Action and Role in Digital Public Health. Eur. J. Public Health.

[3] Rekow E.D. (2020). Digital Dentistry: The New State of the Art—Is It Disruptive or Destructive?. Dent. Mater..

[4] Brod C. (1982). Managing Technostress: Optimizing the Use of Computer Technology. Pers. J..

[5] Ragu-Nathan T., Tarafdar M., Ragu-Nathan B., Ragu-Nathan T., Ragu-Nathan Q. (2008). The Consequences of Technostress for End Users in Organizations: Conceptual Development and Empirical Validation. Inf. Syst. Res..

[6] Tarafdar M., Cooper C., Stich J. (2019). The Technostress Trifecta-techno Eustress, Techno Distress and Design: Theoretical Directions and an Agenda for Research. Inf. Syst. J..

[7] Tarafdar M., Tu Q., Ragu-Nathan B., Ragu-Nathan T. (2007). The Impact of Technostress on Role Stress and Productivity. J. Manag. Inf. Syst..

[8] Golz C., Peter K.A., Zwakhalen S.M.G., Hahn S. (2021). Technostress Among Health Professionals—A Multilevel Model and Group Comparisons between Settings and Professions. Inform. Health Soc. Care.

[9] Raišienė A.G., Jonušauskas S. (2013). Silent Issues of ICT Era: Impact of Techno-Stress to the Work and Life Balance of Employees. Entrep. Sustain. Issues.

[10] Fuglseth A.M., Sørebø Ø. (2014). The Effects of Technostress within the Context of Employee Use of ICT. Comput. Hum. Behav..

[11] Bondanini G., Giorgi G., Ariza-Montes A., Vega-Muñoz A., Andreucci-Annunziata P. (2020). Technostress Dark Side of Technology in the Workplace: A Scientometric Analysis. Int. J. Environ. Res. Public. Health.

[12] Salanova M., Llorens S., Cifre E. (2013). The Dark Side of Technologies: Technostress among Users of Information and Communication Technologies. Int. J. Psychol..

[13] Mark G., Gudith D., Klocke U. (2008). The Cost of Interrupted Work: More Speed and Stress. Proceedings of the SIGCHI conference on Human Factors in Computing Systems.

[14] Gimpel H., Lanzl J., Manner-Romberg T., Nüske N. (2018). Digitaler Stress in Deutschland: Eine Befragung von Erwerbstätigen Zu Belastung Und Beanspruchung Durch Arbeit Mit Digitalen Technologien. Hans-Böckler-Stift..

[15] Tajirian T., Stergiopoulos V., Strudwick G., Sequeira L., Sanches M., Kemp J., Ramamoorthi K., Zhang T., Jankowicz D. (2020). The Influence of Electronic Health Record Use on Physician Burnout: Cross-Sectional Survey. J. Med. Internet Res..

[16] Bahr T.J., Ginsburg S., Wright J.G., Shachak A. (2023). Technostress as Source of Physician Burnout: An Exploration of the Associations between Technology Usage and Physician Burnout. Int. J. Med. Inf..

[17] Dragano N., Lunau T. (2020). Technostress at Work and Mental Health: Concepts and Research Results. Curr. Opin. Psychiatry.

[18] Bernburg M., Gebhardt J.S., Groneberg D.A., Mache S. (2025). Impact of Digitalization in Dentistry on Technostress, Mental Health, and Job Satisfaction: A Quantitative Study. Healthcare.

[19] Petrucci M., Wortz M. (2007). Sampling Und Stichprobe. QUASUS Qualitatives Methodenportal zur Qualitativen Sozial-, Unterrichts-und Schulforschung.

[20] Creswell J.W. (2013). Qualitative Inquiry and Research Design: Choosing among Five Approaches.

[21] Tong A., Sainsbury P., Craig J. (2007). Consolidated Criteria for Reporting Qualitative Research (COREQ): A 32-Item Checklist for Interviews and Focus Groups. Int. J. Qual. Health Care.

[22] Merkens H., Friebertshäuser B., Prengel A. (1997). Stichproben Bei Qualitativen Studien. Handbuch Qualitative Forschungsmethoden in der Erziehungswissenschaft.

[23] Corbin J., Strauss A. (2014). Basics of Qualitative Research: Techniques and Procedures for Developing Grounded Theory.

[24] Helfferich C., Baur N., Blasius J. (2019). Leitfaden- Und Experteninterviews. Handbuch Methoden der empirischen Sozialforschung.

[25] Bogner A., Littig B., Menz W. (2014). Interviews Mit Experten Eine Praxisorientierte Einführung.

[26] Meuser M., Nagel U., Garz D., Kraimer K. (1991). Qualitativ-Empirische Sozialforschung: Konzepte, Methoden, Analysen. Expert Inneninterviews—Vielfach Erprobt, Wenig Bedacht: Ein Beitrag zur Qualitativen Methodendiskussion.

[27] Helfferich C. (2011). Die Qualität Qualitativer Daten. Manual Für Die Durchführung Qualitativer Interviews.

[28] Witzel A., Jüttemann G. (1985). Das Problemzentrierte Interview. Qualitative Forschung in der Psychologie.

[29] Kuckartz U., Rädiker S. (2021). Fokussierte Interviewanalyse Mit MAXQDA Schritt Für Schritt.

[30] Mayring P., Flick U., von Kardoff E., Steinke I. (2012). Qualitative Inhaltsanalyse. Qualitative Forschung. Ein Handbuch.

[31] Mayring P. (2015). Qualitative Inhaltsanalyse. Grundlagen Und Techniken.

[32] Golz C., Peter K.A., Müller T.J., Mutschler J., Zwakhalen S.M., Hahn S. (2021). Technostress and Digital Competence among Health Professionals in Swiss Psychiatric Hospitals: Cross-Sectional Study. JMIR Ment. Health.

[33] Zaresani A., Scott A. (2020). Does Digital Health Technology Improve Physicians’ Job Satisfaction and Work–Life Balance? A Cross-Sectional National Survey and Regression Analysis Using an Instrumental Variable. BMJ Open.

[34] Marsh E., Vallejos E., Spence A. (2022). The Digital Workplace and Its Dark Side: An Integrative Review. Comput. Hum. Behav..

[35] van der Zande M., Gorter R., Aartman I., Wismeijer D. (2015). Adoption and Use of Digital Technologies among General Dental Practitioners in the Netherlands. PLoS ONE.

[36] Jafarpour D., Haricharan P.B., de Souza R.F. (2024). CAD/CAM versus Traditional Complete Dentures: A Systematic Review and Meta-Analysis of Patient- and Clinician-Reported Outcomes and Costs. J. Oral Rehabil..

[37] Pullishery F., Huraib W., Alruhaymi A. (2023). Intraoral Scan Accuracy and Time Efficiency in Implant-Supported Fixed Partial Dentures: A Systematic Review. Cureus.

[38] Mengel R., Candir M., Shiratori K., Flores-de-Jacoby L. (2005). Digital Volume Tomography in the Diagnosis of Periodontal Defects: An in Vitro Study on Native Pig and Human Mandibles. J. Periodontol..

[39] Ben Rehouma M., Geyer T., Kahl T. (2020). Investigating Change Management Based on Participation and Acceptance of IT in the Public Sector: A Mixed Research Study. Int. J. Public Adm. Digit. Age.

[40] Liu C., Cheng T., Cheng C. (2019). Exploring the Factors That Influence Physician Technostress from Using Mobile Electronic Medical Record. Inf. Health Soc. Care.

[41] Heponiemi T., Kujala S., Vainiomäki S., Vehko T., Lääveri T., Vänskä J., Ketola E., Puttonen S., Hyppönen H. (2019). Usability Factors Associated With Physician’s Distress and Information System-Related Stress: Cross-Sectional Survey. JMIR Med. Inf..

[42] Kuek A., Hakkennes S. (2020). Healthcare Staff Digital Literacy Levels and Their Attitudes towards Information. Health Inform. J..

[43] Gimpel H., Lanzl J., Regal C. (2019). Gesund Digital Arbeiten?!. Eine Studie Zu Digitalem Stress in Deutschland.

[44] Sommovigo V., Bernuzzi C., Finstad G.L., Setti I., Gabanelli P., Giorgi G., Fiabane E. (2023). How and When May Technostress Impact Workers’ Psycho-Physical Health and Work-Family Interface? A Study during the COVID-19 Pandemic in Italy. Int. J. Environ. Res. Public. Health.

[45] Bretherton R., Chaoman H., Chipchase S. (2016). A Study to Explore Specific Stressors and Coping Strategies in Primary Dental Care Practice. Br. Dent. J..

[46] Paul M., Maglaras L., Ferrag M., Almomani I. (2023). Digitization of Healthcare Sector: A Study on Privacy and Security Concerns. ICT Express.

[47] Toon M., Collin V., Whitehead P., Reynolds L. (2019). An Analysis of Stress and Burnout in UK General Dental Practitioners: Subdimensions and Causes. Br. Dent. J..

