# Supplementary material for: Digitalization in Dentistry: Dentists’ Perceptions of Digital Stressors and Resources and Their Association with Digital Stress in Germany—A Qualitative Study
Source: Healthcare (Basel). 2025 Jun 17;13(12):1453. doi: 10.3390/healthcare13121453 (PMC12192653; doi:10.3390/healthcare13121453)
Supplement: Supplementary file 1 [file healthcare-13-01453-s001.zip › healthcare-3645461-supplementary.pdf]

Supplementary Materials S1

Supplementary Materials S1.1

The following supplementary material provides additional quotes from the interview responses. They are organized into categories according to chapters.

Table S1. Additional interview quotes.

Category	Citations
**3.3. Experiences with Digital Assistance Systems in Dentistry: A Practice-Based Evaluation**	
3.3.1. Negative Experiences in the Use of Digital Assistance Systems	
General challenges	*"Because there are simply too many tools. There are too many software tools in our software landscape. Infoskop is very unstable and [...] it is often not used because it just doesn't work." (Participant 5, male, age 30-39)*
CAD/CAM/CEREC systems	*"Also, when milling is circular work, the machines operate for about 5-6 hours. So they do take their time. And the technician has to design it as well." (Participant 4, female, age 30-39)*<br>*"She curses regularly because the program sometimes doesn't work. Something freezes then." (Participant 7, female, age 30-39)*<br>*"Actually, in my opinion, the computer program was too precise, because I had to grind down the teeth quite a lot for the program to recognize the parallelism. Whereas I know that the handheld device acknowledges this much sooner and can achieve good results with it." (Participant 7, female, age 30-39)*
Intraoral scanners	*"There was already a case where the camera malfunctioned and didn't take any pictures." (Participant 4, female, age 30-39)*<br>*"For example, with a complete denture, […] initially, we scanned the old denture practically in a bath. But the dental technician cannot match the information then. So these are experiences that you will know for next time, things you need to get right. You have to feel your way into it a bit first." (Participant 4, female, age 30-39)*
Telematics	*"Right now, the new telematics system isn't working. We were really frustrated about it. There are far too many people involved for the system to function. It's extremely, extremely frustrating to figure out what you even need to do and who is responsible. Because everyone says, 'No, that's not my responsibility.' At the moment, it still doesn't seem to be working, and it's very exhausting and time-consuming." (Participant 7, female, age 30-39)*
Practice software	*"And the biggest problems are simply Wi-Fi and fiber optics. […]. But as a practice owner, there's nothing you can do about it. It's just imposed on you […]. Sometimes the network goes down, sometimes there's no signal, and once someone even hacked into Vodafone's line." (Participant 9, female, age 50-59)*

	"Well, sometimes these are technical issues. For example, the device can't connect to the computer, or when I want to make changes in planning, like the fee schedule, and I can't change it." (Participant 7, female, age 30-39)
3.3.2. Positive Experiences in the Use of Digital Assistance Systems	
General experiences	*"But when it does work, I think it really allows a smooth workflow." (Participant 7, female, age 30-39)*<br><br>*"Every software […] must be tailored to individual use. Of course, a practice management software with extensive capabilities can only make the workday easier and lead to a satisfying experience if it is well-configured." (Participant 5, male, age 30-39)*
CAD/CAM/CEREC systems	*"That is a huge advantage, […] the communication with the dental technician. […] Then he sends you a WhatsApp screenshot from his CAD program, and you can provide immediate feedback." (Participant 1, male, age 30-39)*<br><br>*"My boss makes restorations that look truly perfect. […] It's as if the tooth has always been like that." (Participant 8, female, age 20-29)*
Intraoral scanners	*"But basically, I have no problems with the scanner. There's nothing I can say that doesn't work. So it works great." (Participant 4, female, age 30-39)*<br><br>*"I'm thinking of a patient who couldn't be molded at all. He had such a strong gag reflex that it could only be done with a scanner, and it worked wonderfully. […] I wouldn't have been able to do that without the scan." (Participant 3, male, age 50-59)*<br><br>*"And we can overlay the scans directly, which means the program can calculate it at the micrometer level without us having to check with the models whether anything has changed. This is simply a new way to assess it." (Participant 6, female, age 20-29)*<br><br>*"But everything you can do with scanners is huge. Also what the lab can do with the dataset. You no longer have to keep the models; you can use them digitally right away. That's just enormous." (Participant 9, female, age 50-59)*
DVT	*"Or, for example, you have a patient who has a maxillary molar with three roots on the X-ray. After endodontic treatment, they still have complaints, and then a DVT is excellent because such superimpositions can also obscure an apical radiolucency. You can then evaluate each apex individually. Or, for example, for exposure, it's helpful because you don't always know if the tooth is located palatinally or vestibularly. […] Or, for example, with the mandibular second molars and the nerves, you can see how the nerve runs because they are sometimes superimposed." (Participant 6, female, age 20-29)*<br><br>*"No, it actually always has added value. It is always used, especially for implant planning or diagnostics." (Participant 8, female, age 20-29)*<br><br>*"Or, if there is also an indentation in the mandible […] that you just can't see on a 2D image. And of course, it is very helpful to see that." (Participant 7, female, age 30-39)*<br><br>*"In my perception, I have also noticed that it is often helpful with endo-perio lesions to assess the prognosis for the tooth or the extent of furcation involvement." (Participant 8, female, age 20-29)*

Digital X-ray systems	*"I think that's quite good as well, especially when you want to measure the length again during an endo treatment or see what the root looks like." (Participant 2, female, age 20-29)*
Practice software	*"From the very beginning, it was truly a very advanced system, very comprehensive, with many customization options. […] I've only really noticed this after years, how you gradually delve deeper into the possibilities and realize what incredibly great opportunities you have. For that, I would like to highlight 'Evident' because it's a software solution that is at the forefront in terms of the freedoms it offers. You really need to be able to use it and engage with it. If I were to evaluate it, I would say I'm highly satisfied; it definitely deserves a top score." (Participant 3, male, age 50-59)*<br><br>*"It also creates good documentation and provides security that working hours are recorded clearly and accurately." (Participant 3, male, age 50-59)*<br><br>*"There has been a tool that integrates personnel files and time tracking systems. […] For example, this way, the assistants have an overview of their hours, including their overtime and deficits. I think it's great that this is integrated into the dental software. I would like to see more of that. These are things that are very practical and relevant to daily life." (Participant 3, male, age 50-59)*<br><br>*"This is very helpful for me, especially regarding patient records and entries. […] For oral hygiene, all the indices that we had manually noted in the records are now digitized. You can click on them and navigate through. You have everything at a glance." (Participant 8, female, age 20-29)*

3.4. Stress-Inducing Factors: Competitive Pressures and Strategic Adaptions in the Context of Digital Transformation

"I think that as a practice, we have the ambition to keep up with digital progress because we want to stay at the forefront of medical advancements, not necessarily for competitive reasons. For me, this is actually a good sign, as it shows that the practitioner wants to get the best outcomes for the patient." (Participant 8, female, age 20-29)

"It's mainly the patients who have high expectations. You want to meet those expectations, and there is definitely a certain pressure to keep up and deliver." (Participant 7, female, age 30-39)

"You have to be at the top of the Google ratings and have good reviews on Yameda. It's just frustrating. It might put some pressure on you. You feel like you have to keep up with the digital side of things to always look good." (Participant 4, female, age 30-39)

"Those are large clinics in big locations. For my area, I am quite advanced. […] I am actually the one who has done the most in terms of digitalization." (Participant 9, female, age 50-59)

"It doesn't put me under pressure because, as I work in a rural area, it's simply not as necessary here at the moment. If I were working in a city like Berlin, where everyone around me has the latest technology, then it would probably put pressure on me if I didn't have it or couldn't offer it." (Participant 2, female, age 20-29)

"You see what materials they use for fillings and what techniques they have, and you think, 'This is prehistoric! We're lucky, we're modern, we're fast.' That's where you get your motivation and positive feedback for yourself. In day-to-day work, it becomes normal after a while. It's really interesting, though, when you see that others don't have it and are struggling much more than we are." (Participant 1, male, age 30-39)

"Yes and no, because in the end, my hands are tied here. It's not like I could decide to do it this way now. […] If you're working in a private practice, there is some pressure." (Participant 6, female, age 20-29)

3.5. Ethical Challenges in the Use of Digital Assistance Systems in Dental Practices	
3.5.1. Responsibility and Error Attribution in Digital Workflows	
General experiences	*"I think a big problem is that you automatically rely on it. Or you tell yourself, 'The device will handle that,' and then you don't think about it anymore because you have ten other things on your mind. You also no longer have the manufacturing process in focus. So yes, I do believe that this presents a challenge." (Participant 2, female, age 20-29)*<br><br>*"No, I still have to prioritize everything for the patient's well-being and can't just do anything because the computer tells me I need to remove much more material. If I see that the nerve is there, I can't just proceed with that. The patient's welfare always comes first. So, I don't think it leads to any limitations in that regard." (Participant 7, female, age 30-39)*
Intraoral scanner	*"So, you're challenged to work more precisely—that's number one. As a dentist, you're re-sponsible for what you scan. What you don't see, the technician can't see either. This means there's less 'sloppiness.'" (Participant 9, female, age 50-59)*<br><br>*"In such a case, you would rather take a step back and say that the scan needs to be redone." (Participant 4, female, age 30-39)*<br><br>*"If a crown doesn't fit and I've scanned it, I can't just blame the technology and say, 'Look, I overlooked something.' The error has to lie somewhere, and it always comes down to the person who did it. That's just logical." (Participant 9, female, age 50-59)*<br><br>*"Well, scanning is still a manual process. Therefore, the eye and hand of the user are also involved." (Participant 3, male, age 50-59)*
CAD/CAM	*"Especially when the preparation margin isn't entirely clear, it's important to review it together because we've seen it ourselves the best." (Participant 2, female, age 20-29)*<br><br>*"But, and that's the thing, there are often discrepancies between the planning and the clinical model. I believe there's still some development needed in this area. At least for now, it's not the case that you can trust it blindly." (Participant 6, female, age 20-29)*
Implant planning software	*"I'm responsible for ensuring the implants are positioned correctly. I plan the implants digitally myself, and then the technician continues with creating the guide." (Participant 4, female, age 30-39)*<br><br>*"I've always done implants freehand. I still do. But for some cases, I now plan digitally, although I'm still cautious and trying*

it out. I don't rely on it one hundred percent yet. I just need to do it a hundred more times to be able to say, 'It works.' You know, I'll start drilling, then take the guide off to check if it's correct, and then continue drilling. So, I'm still constantly checking along the way." (Participant 4, female, age 30-39)

3.5.2. Broader Ethical Implications: Data Protection, Over-Treatment, and Depersonalization

"But in the lab, the technician only has a standard model because it is ground to a standard specification. That is always the goal. In my opinion, one can only take advantage of the digital advances in this context. But there is still a person sitting in front of it." (Participant 10, female, age 50-59)

"You have to get a signature every time, asking, 'Can I send your data here? Can I use that?' Then you have to archive everything, which also takes time. You also need staff to keep an eye on ensuring that the medical histories are digital, that they are all correct, […]. It's extremely frustrating. And it keeps getting worse, the more digital the work gets." (Participant 1, male, age 30-39)

"When you are digital, you are dependent on so many factors. For example, we once had a cyber incident […]. We actually experienced a cyber attack through a VPN tunnel, and it completely paralyzed our software. This, of course, poses a significant risk. As a practice owner, you then have to invest an enormous amount of money to install many firewalls. This is certainly annoying and costs a lot of money." (Participant 9, female, age 50-59)

"Data protection regulations are always a topic. Germany has a reputation for being a data leak, to be honest. That's always an issue. We can only hope that it remains somewhat vague." (Participant 9, female, age 50-59)

3.6. Support Needs and Learning Methods for Successful Digital Adoption

3.6.1. User-friendliness and Application Support

"Yes, it's always like that for me: everything I can't do, I don't like doing. And when I've practiced things a bit and notice that I'm doing much better, […] then I enjoy doing it much more." (Participant 2, female, age 20-29)

"No, just through 'watch and learn.' It is quite self-explanatory. Ultimately, once you have a general idea of how it works, it's just a matter of practice." (Participant 6, female, age 20-29)

"Well, if you don't have experience yet and want to get some insights, you can always network with colleagues, visit their practices, and see how it works." (participant 3, male, age 50-59)

"So, there's quite a steep learning curve." (Participant 9, female, age 50-59)

"Of course, the more detailed it gets, the more familiar you need to be with it. If there are tasks that aren't done very often, then you notice that the dental assistants struggle a bit if they don't do them regularly. But that's normal. We actually always have instructions for that." (Participant 7, female, age 30-39)

"We don't fully use all the program's capabilities. This is partly because not every employee knows how to operate the system. It's challenging at first to get into it." (Participant 8, female, age 20-29)

3.6.2. Willingness to Learn and Age-Related Differences

"I don't always need to be the first to try something. I have my own experience. If something works well, I don't feel the need to try every new thing. I've seen a lot come and go. It seemed like a good idea, but it didn't always provide relief." (Participant 9, female, age 50-59)

**Participants <50 years old**
"I believe our generation also finds it a bit easier to try things out." (Participant 6, female, age 20-29)
"I'm always a bit curious when we have a new development, wondering how it works and wanting to see it for myself. I think we are a pretty tech-savvy generation, so for us, it's great. For my older colleagues, though, I think it's sometimes not so great—'Another new thing to learn.'" (Participant 2, female, age 20-29)
"These work processes are partly not intuitively designed and are completely difficult for some older employees to understand." (Participant 5, male, age 30-39)
"I believe that since I'm still young, in my late thirties, my motivation is still quite high. For example, the older colleagues around us here in the small town where we live think, 'I have ten or five years left until retirement,' and they have lost their motivation." (Participant 1, male, age 30-39)<br><br>*"I am motivated because I am convinced that digitalization will simplify our lives. Additionally, I am also convinced that we can no longer do without it." (Participant 5, male, age 30-39)*<br><br>*"However, I still have almost thirty working years ahead of me. The motivation has to be high because eventually, others will leave you behind." (Participant 1, male, age 30-39)*<br><br>*"I'm not the first to adopt new things, but I am open to them." (Participant 7, female, age 30-39)*<br><br>*"I'm still quite open and of course I enjoy trying out new things. But there are some aspects where I would generally prefer to work non-digitally. […]I think we often get stuck in what we've learned. However, I hope that this will change a bit and that there will be some solutions that make things easier." (Participant 2, female, age 20-29)*<br><br>*"But everything related to treatment that has an impact and makes sense—that really motivates me. And I show that. I put effort into it, and then I'll invest in the software." (Participant 4, female, age 30-39)*
**Participants >50 years old**
"No one really wants this 'more effort to care.' Colleagues in my age group all feel the same way." (Participant 10, female, age 50-59)<br><br>*"Ten years ago, I used to try new things from time to time. But now I'm the one who says, 'I tried that and didn't get better results.' So I prefer to take the safer route." (Participant 9, female, age 50-59"*<br><br>*"I think it's definitely a generational issue. Young people grow up with it in a completely different way." (Participant 10, female, age 50-59)*<br><br>*"And if you're in a practice with no one who's really tech-savvy, then just let it be. How are you supposed to handle it as a lone operator? But if you have a large practice with young people who are also tech-savvy and like to experiment, then it's fun." (Participant 9, female, age 50-59)*<br><br>*"We have 100 years of medical experience in the practice when you add it all up. Yes, we don't need 3D imaging before an extraction. At least, we haven't needed it so far." (Participant 10, female, age 50-59)*<br><br>*"If I want to acquire something for home, there's no risk of it going wrong. It's just about enhancing my comfort. It either works well or it doesn't; I either use it or I don't. But in the practice, these are core business operations that have consequences if they don't work." (Participant 10, female, age 50-59)*

"You have to be interested in the system, and you need to have a basic understanding of network technology, that's true." (Participant 3, male, age 50-59)<br><br>*"I'm not very open to it because I know that it brings a lot of risks and uncertainties. […] I'm not very open to things where I have no control." (Participant 10, female, age 50-59)*
3.6.3. Support Measures for Sustainable Digital Implementation
"So when I get something new, I first have to learn how to use it. And logically, until I know how to use it, I can't apply it effectively. For that, I need time. And this time comes from two sources: either I give the employee the opportunity to work with this during their working hours, or the employee is motivated enough to possibly take the tool home and work on it in their free time or after work hours." (Participant 5, male, age 30-39)<br><br>*"A stable internet connection is also crucial, and that's not always available everywhere in Germany. In rural areas, it can be particularly challenging. Therefore, they definitely need to promote improvements in this area." (Participant 2, female, age 20-29*<br><br>*"They need to offer more convincing features, more versatile applications—often, these devices only cover certain functions. More flexible integration and follow-up support are also needed. The device just sits there, and there's no follow-up to ensure it's actually being used. It's always additional services you have to pay for and manage yourself." (Participant 10, female, age 50-59)*<br><br>*"If the government wants Germany to become more digital and modern as an industrial nation, they simply need to invest more money and make sure it reaches people. It's no use having a huge fund that isn't utilized just because people don't know it exists. That's where the government needs to start, in my opinion." (Participant 1, male, age 30-39)*<br><br>*"Then the industry shares the risk that what they're leasing or selling isn't the latest technology. Right now, they push out devices at attractive prices, but they're already outdated, and the dentist bears all the risk alone. Nobody wants to be left holding outdated equipment." (Participant 10, female, age 50-59)*<br><br>*"Yes, with leasing, you'd likely have more flexibility. They would have to take it back, so you're not tied to it long-term." (Participant 10, female, age 50-59)*<br><br>*"We had a webinar, but honestly, it didn't help me at all." (Participant 2, female, age 20-29)*<br><br>*"I think that training and further education vary in appeal—some people really enjoy meeting others and learning together. But there are also those who prefer to learn from home, saying, 'I love watching an instructional video and then trying it out on my own.' This can make the learning process a bit easier." (Participant 2, female, age 20-29)*<br><br>*"Integration into university studies, where in practical courses every student would be required to create a CAD/CAM crown." (Participant 8, female, age 20-29)*<br><br>*"This documentation at the 3D level might provide us with better access to the university, scientific thinking, and academic discourse. […] So, this could help us think beyond our own fields—from dentistry to ENT, anatomy, or even psychology—and link these areas more effectively." (Participant 3, male, age 50-59)*

Supplementary Materials S2

Supplementary Materials S2.1

This checklist indicates in which section each item is reported in the manuscript. If N/A is indicated, the manuscript does not provide any statements regarding the item.

Table S2. COREQ checklist (Consolidated Criteria for Reporting Qualitative studies: 32-item checklist)

Item No.	Guide Questions/Description	Reported on Page #
**Domain 1: Research team and reflexivity**		
Personal Characteristics		
1. Interviewer/ facilitator	Which author/s conducted the interview or focus group?	Pg 4
2. Credentials	What were the researcher's credentials? E.g., PhD and MD	Pg 1
3. Occupation	What was their occupation at the time of the study?	Pg 1
4. Gender	Was the researcher male or female?	Pg 1
5. Experience and training	What experience or training did the researcher have?	Pg 5
Relationship with participants		
6. Relationship established	Was a relationship established prior to study commencement?	N/A
7. Participant knowledge of the interviewer	What did the participants know about the researcher? E.g., personal goals and reasons for doing the research?	Pg 5
8. Interviewer characteristics	What characteristics were reported about the interviewer/facilitator? E.g., bias, assumptions, reasons, and interests in the research topic	Pg 5
**Domain 2: Study design**		
Theoretical framework		
9. Methodological orientation and Theory	What methodological orientation was stated to underpin the study? E.g., grounded theory, discourse analysis, ethnography, phenomenology, and content analysis	Pg 2-4
Participant selection		
10. Sampling	How were participants selected? E.g., purposive, convenience, consecutive, and snowball	Pg 5
11. Method of approach	How were participants approached? E.g., face-to-face, telephone, mail, and email	Pg 4
12. Sample size	How many participants were in the study?	Pg 4
13. Non-participation setting	How many people refused to participate or dropped out? Reasons?	N/A
14. Setting of data collection	Where was the data collected? E.g., home, clinic, and workplace	Pg 4
15. Presence of nonparticipants	Was anyone else present besides the participants and researchers?	N/A
16. Description of sample	What are the important characteristics of the sample? E.g., demographic data and date	Pg 3
Data collection		
17. Interview guide	Were questions, prompts, and guides provided by the authors? Was it pilot-tested?	Pg 4
18. Repeat interviews	Were repeat interviews carried out? If yes, how many?	N/A
19. Audio/visual recording	Did the research use audio or visual recording to collect the data?	Pg 4
20. Field notes	Were field notes made during and/or after the interview or focus group?	N/A
21. Duration	What was the duration of the interviews or focus group?	Pg 4

Item No.	Guide Questions/Description	Reported on Page #
22. Data saturation	Was data saturation discussed?	Pg 4
23. Transcripts returned	Were transcripts returned to participants for comment and/or correction?	Pg 4
**Domain 3: Analysis and findings**		
Data analysis		
24. Number of data coders	How many data coders coded the data?	Pg 5
25. Description of the coding tree	Did the authors provide a description of the coding tree?	Pg 5
26. Derivation of themes	Were themes identified in advance or derived from the data?	Pg 5
27. Software	What software, if applicable, was used to manage the data?	Pg 5
28. Participant checking	Did participants provide feedback on the findings?	Pg 5
Reporting		
29. Quotations presented	Were participant quotations presented to illustrate the themes/findings? Was each quotation identified? E.g., participant number	Pg 8-18; Table S1
30. Data and findings consistent	Was there consistency between the data presented and the findings?	Pg 5-22
31. Clarity of major themes	Were major themes clearly presented in the findings?	Pg 5-22
32. Clarity of minor themes	Is there a description of diverse cases or a discussion of minor themes?	Pg 5-22

Tong A, Sainsbury P, Craig J. "Consolidated Criteria for Reporting Qualitative research (COREQ): A 32-Item Checklist for Interviews and Focus Groups." *International Journal for Quality in Health Care*. 2007. Volume 19, Number 6: pp. 349 – 357 7, https://doi.org/10.1093/intqhc/mzm042

Supplementary Materials S3

Supplementary Materials S3.1

This presentation provides a structured overview of the coding system, visualizing its hierarchical relationships. The coding system consists of various categories and subcategories that represent different aspects of the data analysis process.

Table S3. Coding system hierarchical structure.

List of Codes	Frequency
Coding system	894
Citation-worthy text passages	30
Flowers by the wayside	4
Needs assessment	128
User-friendliness of digital tools	13
Comparison with other practices	20
Own willingness to learn	33

Support measures	33
Support, maintenance, and repair	5
Choice of digital assistance systems	13
Desire for more	11
Impacts on work satisfaction	40
Negative long-term effects	15
Positive long-term effects	23
Impacts on strain experience	54
Impairing effects	22
Positive stimulating effects	32
Ethical challenges	31
Error management	6
Responsibility	21
Other ethical questions	4
Impacts on individual demands and resources	102
Quality and aesthetics	23
Treatment experience	11
Potentially increased error-proneness	8
Reduced error-proneness	20
Acceleration	30
No time-savings	10
Impacts on workload experience	97
Work facilitation	45
Additional workload	28
Task delegation	14
Consistent workload experience	10
Subjective usage assessment	102
Economic aspects	32
Negative experience reports	41
Positive experience reports	29
Other	9
Consideration of the patient perspective	64

Effects on patient communication	18
Patient feedback	28
Patient education	12
Presence on the practice website	6
Usage behavior	119
Frequency of use	29
Digital assistance systems used in practice	45
Application of digital assistance systems	45
Person-specific information	87
Working hours	22
Specialization/treatment spectrum	14
Career path	19
Employment contract	12
Daily practice routine	7
Age	10
Educational background	3
Practice-specific information	27
Dental laboratory	12
Practice structure	15
